# Trichlorido[4-meth­oxy-2,6-bis­(2-pyrimidin-2-yl-κ*N*)phenyl-κ*C* ^1^]platinum(IV) acetonitrile monosolvate

**DOI:** 10.1107/S1600536812036410

**Published:** 2012-08-25

**Authors:** Yang Wu, Dahai Xie, Dengqing Zhang, Xianying Li, Wusong Jin

**Affiliations:** a College of Chemistry, Chemical Engineering and Biotechnology, Donghua University, 2999 North Renmin Road, Songjiang, Shanghai 201620, People’s Republic of China; b School of Environmental Science and Engineering, Donghua University, 2999 North Renmin Road, Songjiang, Shanghai 201620, People’s Republic of China

## Abstract

In the title complex, [Pt(C_15_H_11_N_4_O)Cl_3_]·CH_3_CN, the Pt^IV^ ion adopts a distorted octa­hedral coordination geometry defined by a tridentate cyclo­metalated NCN ligand and three Cl atoms. In the crystal, individual mol­ecules are aggregated into a three-dimensional network by C—H⋯Cl hydrogen-bonding inter­actions and π–π stacking inter­actions between the tridentate ligands, the shortest ring centroid–centroid distance being 3.613 Å.

## Related literature

For general background to the chemistry of the tridentate NCN ligand and its complexes, see: Williams (2009 ▶); Wang *et al.* (2010 ▶); Chen *et al.* (2009 ▶); Lu *et al.* (2009 ▶). For the synthesis of related ligand, see: Avitia *et al.* (2011 ▶); Wakioka *et al.* (2010 ▶). For Pt^II^ complexes with tridentate NCN ligands, see: Kozhevnikov *et al.* (2008 ▶); Tam *et al.* (2011 ▶). For Pt—Cl bond lengths in other Pt^IV^ complexes, see: Bagchi *et al.* (2007 ▶); Bokach *et al.* (2012 ▶). For details of the preparation, see: Cardenas & Echavarren (1999 ▶).
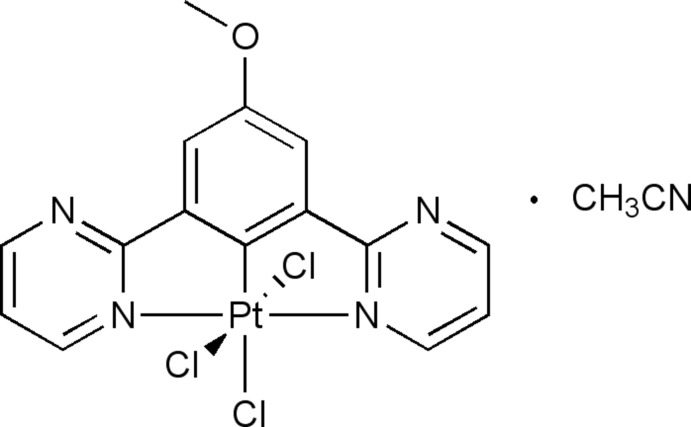


## Experimental

### 

#### Crystal data

[Pt(C_15_H_11_N_4_O)Cl_3_]·C_2_H_3_N
*M* *_r_* = 605.77
Triclinic, 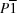

*a* = 8.5739 (8) Å
*b* = 10.3371 (10) Å
*c* = 12.6610 (12) Å
α = 68.955 (2)°
β = 80.033 (2)°
γ = 70.619 (2)°
*V* = 986.09 (16) Å^3^
*Z* = 2
Mo *K*α radiation
μ = 7.54 mm^−1^
*T* = 293 K
0.21 × 0.16 × 0.13 mm

#### Data collection

Bruker SMART CCD area-detector diffractometer
Absorption correction: multi-scan (*SADABS*; Bruker, 2000 ▶) *T*_min_ = 0.346, *T*_max_ = 1.000
5788 measured reflections
3666 independent reflections
3442 reflections with *I* > 2σ(*I*)
*R*_int_ = 0.026

#### Refinement

*R*[*F* ^2^ > 2σ(*F* ^2^)] = 0.033
*wR*(*F* ^2^) = 0.085
*S* = 1.04
3666 reflections
246 parameters
H-atom parameters constrained
Δρ_max_ = 1.63 e Å^−3^
Δρ_min_ = −1.61 e Å^−3^

### 

Data collection: *SMART* (Bruker, 2000 ▶); cell refinement: *SAINT* (Bruker, 2000 ▶); data reduction: *SAINT*; program(s) used to solve structure: *SHELXS97* (Sheldrick, 2008 ▶); program(s) used to refine structure: *SHELXL97* (Sheldrick, 2008 ▶); molecular graphics: *SHELXTL* (Sheldrick, 2008 ▶); software used to prepare material for publication: *SHELXTL*.

## Supplementary Material

Crystal structure: contains datablock(s) I, global. DOI: 10.1107/S1600536812036410/hp2041sup1.cif

Structure factors: contains datablock(s) I. DOI: 10.1107/S1600536812036410/hp2041Isup2.hkl

Additional supplementary materials:  crystallographic information; 3D view; checkCIF report

## Figures and Tables

**Table 1:** Selected bond lengths (Å)

Pt1—C5	1.944 (5)
Pt1—N3	2.038 (4)
Pt1—N1	2.046 (4)
Pt1—Cl3	2.3018 (16)
Pt1—Cl2	2.3528 (15)
Pt1—Cl1	2.4160 (15)

**Table 2:** Hydrogen-bond geometry (Å, °)

*D*—H⋯*A*	*D*—H	H⋯*A*	*D*⋯*A*	*D*—H⋯*A*
C14—H14⋯Cl1^i^	0.93	2.61	3.330 (6)	135
C4—H4⋯Cl2^ii^	0.93	2.84	3.680 (6)	151
C12—H12⋯Cl3^iii^	0.93	2.78	3.509 (6)	136

Symmetry codes: (i) 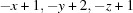; (ii) 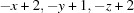; (iii) 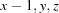.

**Fig. 1.**
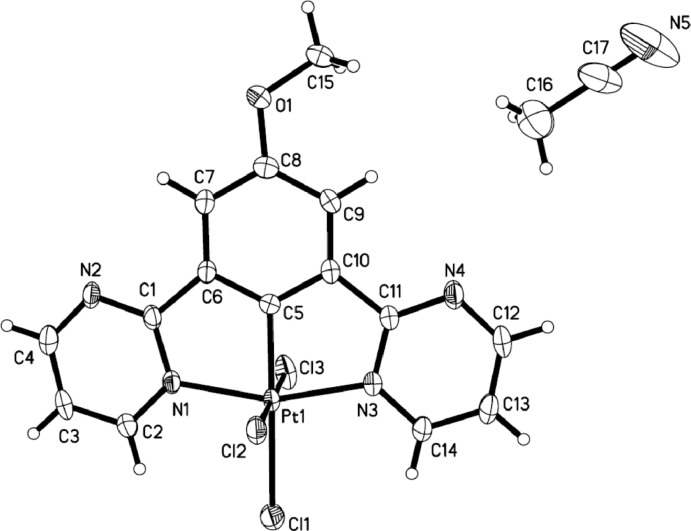
Perspective view of the title compound with displacement ellipsoids at the 30% probability level.

**Fig. 2.**
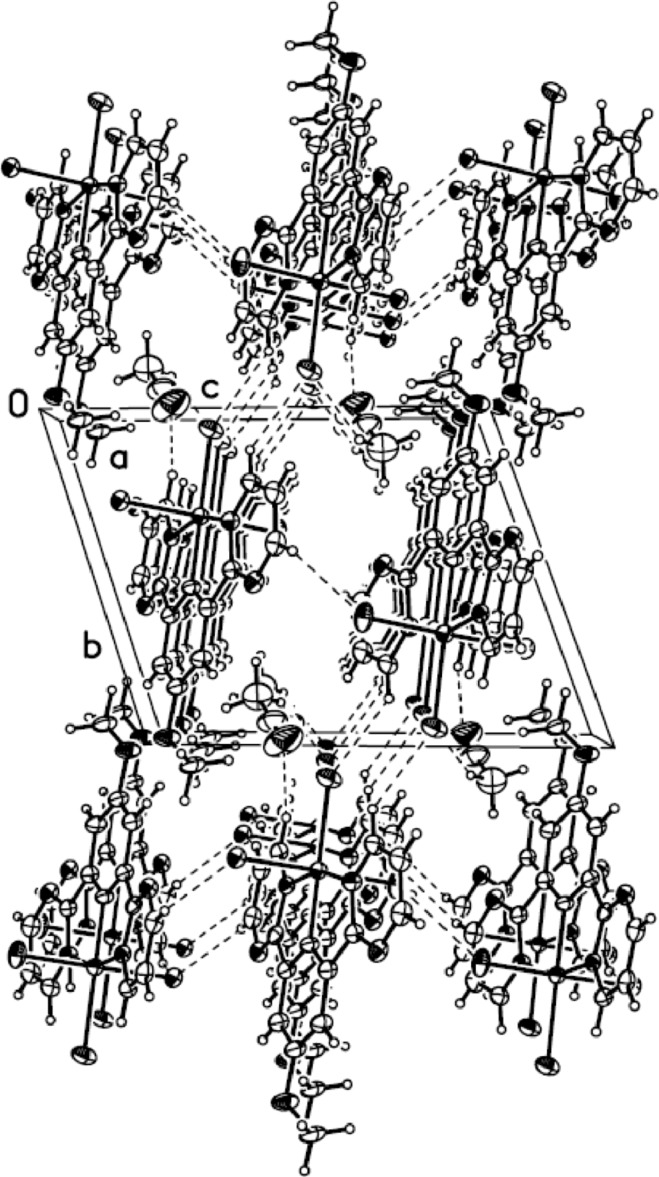
A view of the unit-cell contents of the title complex.

## supplementary crystallographic information

### Comment

Square planar Pt^II^ complexes with tridentate cyclometalating NCN ligands [*e.g.*, *m*-di(2-pyridinyl)benzene (dpb) derivatives (Wang *et al.*, 2010)] have attracted a great attention due to their potential application in wide ranges such as chemosensors, luminescent materials, as well as photovoltaic cells (Williams 2009; Chen *et al.*, 2009; Lu *et al.*, 2009). Recently, some Pt^II^ complex with novel tridentate NCN ligands have been reported (Tam *et al.*, 2011; Kozhevnikov *et al.*, 2008). In this point of view, we develop a novel tridentate NCN ligand (Avitia *et al.*, 2011; Wakioka *et al.*, 2010), 1,3-di(2'-pyrimidyl)-5-methoxybenzene. Interestingly, in an attempt to prepare square Pt^II^ complex by the reaction of K_2_PtCl_4_ with newly synthesized ligand, the neutral Pt^IV^ complex, [Pt(C_15_H_11_N_4_O)Cl_3_].CH_3_CN, was unexpected obtained as a byproduct along with the Pt^II^ complex, [Pt(C_15_H_11_N_4_O)Cl].

The asymmetric unit of the title compound, contains a neutral Pt^IV^ complex and one acetonitrile molecule (Fig. 1). The central platinum(IV) atom is coordinated by two nitrogen atoms and one carbon atom from tridentate ligand, and three chlorine atoms forming a distorted octahedral geometry. The angles C5—Pt1—Cl3, N3—Pt1—Cl3, N1—Pt1—Cl3, C5—Pt1—Cl2, Cl3—Pt1—Cl1, N3—Pt1—Cl2, N1—Pt1—Cl2 and Cl2—Pt1—Cl1 (88.14 (13)—92.288 (13)°, Table 1) are very close to the ideal 90°, the deviation from 90° of the angles C5—Pt1—N3 (80.3 (2)°) and C5—Pt1—N1 (80.7 (2)°) are owing to the formation of five-membered chelating rings. The bond lengths of Pt–N1, Pt–C5, Pt–N3, and Pt–Cl1 (Table 1) are similar to the literature reported Pt(II)dpbCl complexes (Wang *et al.*, 2010). The bond lengths of Pt–Cl2 and Pt–Cl3 (Table 1) resemble those in other Pt^IV^ complexes, which have been published (Bagchi *et al.*, 2007; Bokach *et al.*, 2012). The title complex packed as head-to-tail dimers in the crystal, each molecular unit of the dimer related to the other by a center of inversion. They are further connected into three-dimension crystal structure *via* C—H···Cl hydrogen-bonding interactions and *π–π* stacking interactions between tridentate ligands, the shortest ring centroid-centroid distance is 3.613 Å (Table 2).

### Experimental

A mixture of 1,3-di(2'-pyrimidyl)-5-methoxybenzene 200 mg (0.756 mmol) and K_2_PtCl_4_ 314 mg (0.756 mmol) in CH_3_CN/H_2_O (*v*/*v*, 1/1, 60 ml) was stirred under reflux for 24 h in an argon atmosphere (Cardenas & Echavarren,, 1999). The resulted red solution was evaporated and the residue was extracted with CH_2_Cl_2_. The title complex was separated by flash column chromatography (SiO_2_, CH_2_Cl_2_ as eluent) from the mixture. The single-crystal was obtained by slow evaporation of an acetonitrile solution of the title complex.

### Refinement

All H atoms were placed geometrically and treated as riding on their parent atoms with C—H = 0.96 (methyl) Å [U iso (H) = 1.5U eq (C)], and C—H = 0.93 (aromatic) Å [U iso (H) = 1.2U eq (C)].

### Crystal data

**Table:**

[Pt(C_15_H_11_N_4_O)Cl_3_]·C_2_H_3_N	*Z* = 2
*M**_r_* = 605.77	*F*(000) = 576
Triclinic, *P*1	*D*_x_ = 2.040 Mg m^−^^3^
Hall symbol: -P 1	Mo *K*α radiation, λ = 0.71073 Å
*a* = 8.5739 (8) Å	Cell parameters from 3770 reflections
*b* = 10.3371 (10) Å	θ = 4.6–56.4°
*c* = 12.6610 (12) Å	µ = 7.54 mm^−^^1^
α = 68.955 (2)°	*T* = 293 K
β = 80.033 (2)°	Prismatic, red
γ = 70.619 (2)°	0.21 × 0.16 × 0.13 mm
*V* = 986.09 (16) Å^3^	

### Data collection

**Table:**

Bruker SMART CCD area-detector diffractometer	3666 independent reflections
Radiation source: fine-focus sealed tube	3442 reflections with *I* > 2σ(*I*)
Graphite monochromator	*R*_int_ = 0.026
phi and ω scans	θ_max_ = 25.5°, θ_min_ = 1.7°
Absorption correction: multi-scan (*SADABS*; Bruker, 2000)	*h* = −9→10
*T*_min_ = 0.346, *T*_max_ = 1.000	*k* = −12→11
5788 measured reflections	*l* = −15→9

### Refinement

**Table:**

Refinement on *F*^2^	Primary atom site location: structure-invariant direct methods
Least-squares matrix: full	Secondary atom site location: difference Fourier map
*R*[*F*^2^ > 2σ(*F*^2^)] = 0.033	Hydrogen site location: inferred from neighbouring sites
*wR*(*F*^2^) = 0.085	H-atom parameters constrained
*S* = 1.04	*w* = 1/[σ^2^(*F*_o_^2^) + (0.0539*P*)^2^] where *P* = (*F*_o_^2^ + 2*F*_c_^2^)/3
3666 reflections	(Δ/σ)_max_ = 0.002
246 parameters	Δρ_max_ = 1.63 e Å^−^^3^
0 restraints	Δρ_min_ = −1.61 e Å^−^^3^

### Special details

**Table:**

Geometry. All e.s.d.'s (except the e.s.d. in the dihedral angle between two l.s. planes) are estimated using the full covariance matrix. The cell e.s.d.'s are taken into account individually in the estimation of e.s.d.'s in distances, angles and torsion angles; correlations between e.s.d.'s in cell parameters are only used when they are defined by crystal symmetry. An approximate (isotropic) treatment of cell e.s.d.'s is used for estimating e.s.d.'s involving l.s. planes.
Refinement. Refinement of *F*^2^ against ALL reflections. The weighted *R*-factor *wR* and goodness of fit *S* are based on *F*^2^, conventional *R*-factors *R* are based on *F*, with *F* set to zero for negative *F*^2^. The threshold expression of *F*^2^ > σ(*F*^2^) is used only for calculating *R*-factors(gt) *etc*. and is not relevant to the choice of reflections for refinement. *R*-factors based on *F*^2^ are statistically about twice as large as those based on *F*, and *R*- factors based on ALL data will be even larger.

### Fractional atomic coordinates and isotropic or equivalent isotropic displacement parameters (Å^2^)

**Table:**

	*x*	*y*	*z*	*U*_iso_*/*U*_eq_	
Pt1	0.69455 (2)	0.665972 (19)	0.713419 (15)	0.03224 (10)	
Cl1	0.6637 (2)	0.92214 (17)	0.62623 (15)	0.0597 (5)	
Cl2	0.52290 (17)	0.70939 (16)	0.87099 (12)	0.0391 (3)	
Cl3	0.8607 (2)	0.6190 (2)	0.55999 (15)	0.0585 (4)	
N1	0.9051 (5)	0.6081 (5)	0.7956 (4)	0.0329 (9)	
N2	1.1004 (5)	0.4043 (5)	0.9078 (4)	0.0388 (10)	
N3	0.5022 (5)	0.6499 (5)	0.6493 (4)	0.0371 (10)	
N4	0.3832 (6)	0.4766 (6)	0.6415 (4)	0.0440 (11)	
N5	0.0658 (16)	0.0090 (12)	0.7024 (13)	0.160 (5)	
O1	0.7849 (6)	0.0280 (4)	0.9438 (4)	0.0601 (13)	
C1	0.9599 (6)	0.4644 (6)	0.8559 (4)	0.0356 (11)	
C2	0.9942 (6)	0.6958 (6)	0.7895 (4)	0.0374 (12)	
H2	0.9576	0.7943	0.7494	0.045*	
C3	1.1408 (7)	0.6384 (7)	0.8433 (5)	0.0443 (14)	
H3	1.2042	0.6970	0.8410	0.053*	
C4	1.1898 (7)	0.4928 (7)	0.9001 (5)	0.0453 (14)	
H4	1.2896	0.4530	0.9351	0.054*	
C5	0.7221 (7)	0.4595 (5)	0.7844 (5)	0.0355 (12)	
C6	0.8542 (6)	0.3786 (6)	0.8534 (5)	0.0368 (11)	
C7	0.8712 (7)	0.2321 (6)	0.9065 (5)	0.0444 (13)	
H7	0.9557	0.1741	0.9551	0.053*	
C8	0.7599 (8)	0.1731 (6)	0.8860 (5)	0.0451 (13)	
C9	0.6325 (7)	0.2562 (6)	0.8115 (5)	0.0442 (13)	
H9	0.5622	0.2138	0.7965	0.053*	
C10	0.6146 (7)	0.4025 (6)	0.7612 (5)	0.0381 (12)	
C11	0.4924 (6)	0.5123 (6)	0.6806 (5)	0.0360 (11)	
C12	0.2798 (7)	0.5850 (7)	0.5684 (5)	0.0483 (14)	
H12	0.2031	0.5635	0.5387	0.058*	
C13	0.2806 (8)	0.7262 (8)	0.5348 (5)	0.0498 (15)	
H13	0.2053	0.7991	0.4846	0.060*	
C14	0.3954 (7)	0.7570 (6)	0.5772 (5)	0.0422 (13)	
H14	0.3991	0.8519	0.5560	0.051*	
C15	0.7049 (9)	−0.0469 (6)	0.9062 (7)	0.0591 (17)	
H15A	0.5871	−0.0096	0.9179	0.089*	
H15B	0.7401	−0.1485	0.9485	0.089*	
H15C	0.7336	−0.0331	0.8270	0.089*	
C16	0.2570 (14)	0.1329 (11)	0.7295 (10)	0.100 (3)	
H16A	0.2050	0.2357	0.7068	0.150*	
H16B	0.3587	0.1123	0.6845	0.150*	
H16C	0.2800	0.0975	0.8081	0.150*	
C17	0.1486 (13)	0.0629 (10)	0.7132 (9)	0.092 (3)	

### Atomic displacement parameters (Å^2^)

**Table:**

	*U*^11^	*U*^22^	*U*^33^	*U*^12^	*U*^13^	*U*^23^
Pt1	0.03207 (14)	0.03471 (14)	0.03470 (14)	−0.01178 (9)	−0.01267 (9)	−0.01011 (9)
Cl1	0.0748 (11)	0.0444 (8)	0.0617 (10)	−0.0267 (8)	−0.0317 (8)	0.0017 (7)
Cl2	0.0331 (6)	0.0485 (8)	0.0416 (7)	−0.0113 (6)	−0.0103 (5)	−0.0183 (6)
Cl3	0.0460 (8)	0.0979 (13)	0.0518 (9)	−0.0305 (9)	0.0021 (7)	−0.0415 (9)
N1	0.033 (2)	0.038 (2)	0.032 (2)	−0.0095 (18)	−0.0098 (17)	−0.0137 (18)
N2	0.033 (2)	0.048 (3)	0.038 (2)	−0.008 (2)	−0.0151 (18)	−0.015 (2)
N3	0.035 (2)	0.040 (2)	0.041 (2)	−0.0109 (19)	−0.0124 (19)	−0.012 (2)
N4	0.037 (2)	0.054 (3)	0.049 (3)	−0.011 (2)	−0.014 (2)	−0.024 (2)
N5	0.174 (11)	0.130 (9)	0.227 (14)	−0.102 (9)	0.068 (10)	−0.101 (9)
O1	0.077 (3)	0.035 (2)	0.078 (3)	−0.019 (2)	−0.037 (3)	−0.012 (2)
C1	0.035 (3)	0.042 (3)	0.036 (3)	−0.008 (2)	−0.010 (2)	−0.020 (2)
C2	0.036 (3)	0.042 (3)	0.040 (3)	−0.016 (2)	−0.005 (2)	−0.014 (2)
C3	0.040 (3)	0.066 (4)	0.044 (3)	−0.026 (3)	−0.006 (2)	−0.027 (3)
C4	0.031 (3)	0.064 (4)	0.045 (3)	−0.009 (3)	−0.010 (2)	−0.025 (3)
C5	0.038 (3)	0.027 (2)	0.043 (3)	−0.008 (2)	−0.012 (2)	−0.011 (2)
C6	0.034 (3)	0.037 (3)	0.043 (3)	−0.006 (2)	−0.015 (2)	−0.015 (2)
C7	0.048 (3)	0.040 (3)	0.048 (3)	−0.008 (3)	−0.024 (3)	−0.014 (2)
C8	0.050 (3)	0.032 (3)	0.056 (4)	−0.009 (2)	−0.011 (3)	−0.017 (2)
C9	0.046 (3)	0.039 (3)	0.057 (4)	−0.017 (2)	−0.018 (3)	−0.016 (3)
C10	0.037 (3)	0.041 (3)	0.042 (3)	−0.012 (2)	−0.013 (2)	−0.014 (2)
C11	0.033 (3)	0.040 (3)	0.040 (3)	−0.012 (2)	−0.010 (2)	−0.014 (2)
C12	0.036 (3)	0.069 (4)	0.052 (4)	−0.021 (3)	−0.010 (3)	−0.026 (3)
C13	0.042 (3)	0.061 (4)	0.048 (3)	−0.011 (3)	−0.021 (3)	−0.014 (3)
C14	0.043 (3)	0.043 (3)	0.042 (3)	−0.013 (2)	−0.019 (2)	−0.008 (2)
C15	0.071 (4)	0.032 (3)	0.083 (5)	−0.014 (3)	−0.025 (4)	−0.022 (3)
C16	0.088 (6)	0.098 (7)	0.120 (8)	−0.039 (5)	−0.007 (6)	−0.033 (6)
C17	0.100 (7)	0.063 (5)	0.118 (8)	−0.041 (5)	0.021 (6)	−0.032 (5)

### Geometric parameters (Å, º)

**Table:**

Pt1—C5	1.944 (5)	C4—H4	0.9300
Pt1—N3	2.038 (4)	C5—C10	1.364 (8)
Pt1—N1	2.046 (4)	C5—C6	1.391 (7)
Pt1—Cl3	2.3018 (16)	C6—C7	1.387 (8)
Pt1—Cl2	2.3528 (15)	C7—C8	1.391 (8)
Pt1—Cl1	2.4160 (15)	C7—H7	0.9300
N1—C2	1.342 (7)	C8—C9	1.405 (8)
N1—C1	1.363 (7)	C9—C10	1.380 (8)
N2—C1	1.324 (6)	C9—H9	0.9300
N2—C4	1.345 (8)	C10—C11	1.475 (7)
N3—C14	1.337 (7)	C12—C13	1.368 (9)
N3—C11	1.360 (7)	C12—H12	0.9300
N4—C11	1.334 (7)	C13—C14	1.368 (8)
N4—C12	1.334 (8)	C13—H13	0.9300
N5—C17	1.088 (14)	C14—H14	0.9300
O1—C8	1.373 (7)	C15—H15A	0.9600
O1—C15	1.422 (8)	C15—H15B	0.9600
C1—C6	1.474 (7)	C15—H15C	0.9600
C2—C3	1.381 (8)	C16—C17	1.431 (14)
C2—H2	0.9300	C16—H16A	0.9600
C3—C4	1.366 (9)	C16—H16B	0.9600
C3—H3	0.9300	C16—H16C	0.9600
			
C5—Pt1—N3	80.3 (2)	C7—C6—C1	129.5 (5)
C5—Pt1—N1	80.7 (2)	C5—C6—C1	113.2 (5)
N3—Pt1—N1	160.70 (19)	C6—C7—C8	119.1 (5)
C5—Pt1—Cl3	89.32 (18)	C6—C7—H7	120.5
N3—Pt1—Cl3	88.69 (14)	C8—C7—H7	120.5
N1—Pt1—Cl3	88.14 (13)	O1—C8—C7	114.8 (5)
C5—Pt1—Cl2	89.57 (17)	O1—C8—C9	122.9 (5)
N3—Pt1—Cl2	90.54 (14)	C7—C8—C9	122.3 (5)
N1—Pt1—Cl2	92.28 (13)	C10—C9—C8	118.0 (5)
Cl3—Pt1—Cl2	178.74 (5)	C10—C9—H9	121.0
C5—Pt1—Cl1	179.24 (16)	C8—C9—H9	121.0
N3—Pt1—Cl1	100.49 (13)	C5—C10—C9	118.9 (5)
N1—Pt1—Cl1	98.58 (13)	C5—C10—C11	112.4 (5)
Cl3—Pt1—Cl1	90.63 (7)	C9—C10—C11	128.6 (5)
Cl2—Pt1—Cl1	90.48 (6)	N4—C11—N3	123.5 (5)
C2—N1—C1	119.4 (4)	N4—C11—C10	121.6 (5)
C2—N1—Pt1	126.1 (4)	N3—C11—C10	114.8 (5)
C1—N1—Pt1	114.4 (3)	N4—C12—C13	123.4 (5)
C1—N2—C4	116.8 (5)	N4—C12—H12	118.3
C14—N3—C11	119.1 (5)	C13—C12—H12	118.3
C14—N3—Pt1	126.8 (4)	C12—C13—C14	118.1 (6)
C11—N3—Pt1	114.0 (3)	C12—C13—H13	120.9
C11—N4—C12	116.2 (5)	C14—C13—H13	120.9
C8—O1—C15	117.5 (5)	N3—C14—C13	119.6 (6)
N2—C1—N1	123.3 (5)	N3—C14—H14	120.2
N2—C1—C6	122.2 (5)	C13—C14—H14	120.2
N1—C1—C6	114.4 (4)	O1—C15—H15A	109.5
N1—C2—C3	119.4 (5)	O1—C15—H15B	109.5
N1—C2—H2	120.3	H15A—C15—H15B	109.5
C3—C2—H2	120.3	O1—C15—H15C	109.5
C4—C3—C2	118.0 (5)	H15A—C15—H15C	109.5
C4—C3—H3	121.0	H15B—C15—H15C	109.5
C2—C3—H3	121.0	C17—C16—H16A	109.5
N2—C4—C3	123.1 (5)	C17—C16—H16B	109.5
N2—C4—H4	118.5	H16A—C16—H16B	109.5
C3—C4—H4	118.5	C17—C16—H16C	109.5
C10—C5—C6	124.2 (5)	H16A—C16—H16C	109.5
C10—C5—Pt1	118.4 (4)	H16B—C16—H16C	109.5
C6—C5—Pt1	117.3 (4)	N5—C17—C16	179.0 (14)
C7—C6—C5	117.3 (5)		
			
C5—Pt1—N1—C2	176.2 (5)	Cl2—Pt1—C5—C6	−90.5 (4)
N3—Pt1—N1—C2	167.2 (5)	Cl1—Pt1—C5—C6	4 (14)
Cl3—Pt1—N1—C2	86.5 (4)	C10—C5—C6—C7	−3.6 (9)
Cl2—Pt1—N1—C2	−94.6 (4)	Pt1—C5—C6—C7	178.3 (4)
Cl1—Pt1—N1—C2	−3.8 (4)	C10—C5—C6—C1	174.7 (5)
C5—Pt1—N1—C1	0.1 (4)	Pt1—C5—C6—C1	−3.4 (7)
N3—Pt1—N1—C1	−8.9 (7)	N2—C1—C6—C7	4.6 (9)
Cl3—Pt1—N1—C1	−89.6 (4)	N1—C1—C6—C7	−178.6 (6)
Cl2—Pt1—N1—C1	89.2 (4)	N2—C1—C6—C5	−173.4 (5)
Cl1—Pt1—N1—C1	−179.9 (3)	N1—C1—C6—C5	3.4 (7)
C5—Pt1—N3—C14	−178.9 (5)	C5—C6—C7—C8	1.8 (9)
N1—Pt1—N3—C14	−169.9 (5)	C1—C6—C7—C8	−176.2 (6)
Cl3—Pt1—N3—C14	−89.3 (5)	C15—O1—C8—C7	−164.5 (6)
Cl2—Pt1—N3—C14	91.7 (5)	C15—O1—C8—C9	15.3 (10)
Cl1—Pt1—N3—C14	1.1 (5)	C6—C7—C8—O1	−178.9 (6)
C5—Pt1—N3—C11	−2.8 (4)	C6—C7—C8—C9	1.2 (10)
N1—Pt1—N3—C11	6.2 (8)	O1—C8—C9—C10	177.5 (6)
Cl3—Pt1—N3—C11	86.8 (4)	C7—C8—C9—C10	−2.7 (9)
Cl2—Pt1—N3—C11	−92.3 (4)	C6—C5—C10—C9	2.2 (9)
Cl1—Pt1—N3—C11	177.2 (4)	Pt1—C5—C10—C9	−179.8 (4)
C4—N2—C1—N1	0.8 (8)	C6—C5—C10—C11	−176.8 (5)
C4—N2—C1—C6	177.2 (5)	Pt1—C5—C10—C11	1.3 (7)
C2—N1—C1—N2	−1.5 (8)	C8—C9—C10—C5	1.0 (9)
Pt1—N1—C1—N2	174.9 (4)	C8—C9—C10—C11	179.8 (6)
C2—N1—C1—C6	−178.2 (5)	C12—N4—C11—N3	−0.3 (8)
Pt1—N1—C1—C6	−1.9 (6)	C12—N4—C11—C10	−179.5 (5)
C1—N1—C2—C3	0.7 (8)	C14—N3—C11—N4	1.4 (8)
Pt1—N1—C2—C3	−175.2 (4)	Pt1—N3—C11—N4	−175.0 (4)
N1—C2—C3—C4	0.7 (8)	C14—N3—C11—C10	−179.4 (5)
C1—N2—C4—C3	0.7 (8)	Pt1—N3—C11—C10	4.2 (6)
C2—C3—C4—N2	−1.5 (9)	C5—C10—C11—N4	175.7 (5)
N3—Pt1—C5—C10	0.7 (5)	C9—C10—C11—N4	−3.2 (9)
N1—Pt1—C5—C10	−176.3 (5)	C5—C10—C11—N3	−3.6 (7)
Cl3—Pt1—C5—C10	−88.0 (5)	C9—C10—C11—N3	177.5 (6)
Cl2—Pt1—C5—C10	91.4 (5)	C11—N4—C12—C13	−0.9 (9)
Cl1—Pt1—C5—C10	−175 (25)	N4—C12—C13—C14	1.1 (10)
N3—Pt1—C5—C6	178.9 (5)	C11—N3—C14—C13	−1.2 (9)
N1—Pt1—C5—C6	1.9 (4)	Pt1—N3—C14—C13	174.7 (5)
Cl3—Pt1—C5—C6	90.1 (4)	C12—C13—C14—N3	0.0 (9)

### Hydrogen-bond geometry (Å, º)

**Table:**

*D*—H···*A*	*D*—H	H···*A*	*D*···*A*	*D*—H···*A*
C14—H14···Cl1^i^	0.93	2.61	3.330 (6)	135
C4—H4···Cl2^ii^	0.93	2.84	3.680 (6)	151
C12—H12···Cl3^iii^	0.93	2.78	3.509 (6)	136

Symmetry codes: (i) −*x*+1, −*y*+2, −*z*+1; (ii) −*x*+2, −*y*+1, −*z*+2; (iii) *x*−1, *y*, *z*.
